# Lung ultrasound versus chest radiography for the detection of early postoperative pulmonary complications in children undergoing cardiac surgery

**DOI:** 10.1007/s00431-026-07353-z

**Published:** 2026-09-09

**Authors:** Nehad A. Bakry, Mervat G. Mansour, Heba A. Ali, Ahmed M. Abouelhoda, Mennatullah I. Abdelhamid, Beshoy A. Ebrahim

**Affiliations:** 1 Pediatrics Department, Faculty of Medicine, Ain Shams University, Cairo, Egypt; 2 Radiology Department, Faculty of Medicine, Ain Shams University, Cairo, Egypt

**Keywords:** Lung ultrasound, Chest radiography, Postoperative pulmonary complications, Pediatric cardiac surgery, Extubation outcome, Point-of-care ultrasound

## Abstract

Pulmonary complications are a major cause of morbidity following congenital heart surgery in children. This pilot study evaluated detection rates of point-of-care lung ultrasound (LUS) compared with chest X-ray (CXR) for early identification of postoperative pulmonary complications in pediatric cardiac surgery patients. This prospective observational pilot study enrolled 30 pediatric patients (aged < 4 years) undergoing congenital heart surgery (RACHS-1 categories 1–3) at a single tertiary center (March 2024–March 2025). LUS using a standardized 12-zone protocol was compared with bedside CXR at three assessment timepoints: within 6 h postoperatively (T1), pre-extubation (T2), and post-extubation (T3). Diagnostic agreement was assessed by Cohen’s kappa. Clinical outcomes were analyzed using multivariable logistic regression. LUS and CXR showed equivalent detection of pulmonary congestion at T1 (63.3%; *p* = 1.000). LUS detected higher rates of pleural effusion (40.0% vs. 6.7%; *p* = 0.002) and consolidation (56.7% vs. 33.3%; *p* = 0.069) at T1. Extubation failure occurred in 16.7% (*n* = 5) and was associated with prolonged ICU stay, longer ventilation, and greater inotropic support (all *p* ≤ 0.040). On multivariable logistic regression, longer inotropic support duration was associated with lower odds of successful extubation (exploratory analysis, OR 0.52, *p* = 0.009); imaging variables were not significant (all *p* > 0.27); findings warrant cautious interpretation given the small sample.

*Conclusion*: In this pilot cohort, LUS demonstrated higher detection rates for early postoperative pleural effusion and consolidation compared with CXR, supporting its role as a complementary bedside tool. Extubation outcomes were primarily driven by hemodynamic factors. Larger prospective studies are needed to confirm these preliminary findings.

**Table:** 

**What is Known:** •* Postoperative pulmonary complications occur in up to 76% of children after cardiac surgery and prolong mechanical ventilation and ICU stay.* • *Chest X-ray is the standard bedside imaging tool but has limited sensitivity for early parenchymal and pleural pathology.*	
**What is New:** • *Lung ultrasound detected pleural effusion and consolidation at higher rates than CXR across all three postoperative timepoints.* • *Serial bedside LUS enabled dynamic longitudinal monitoring of complication resolution over 11 postoperative days, beyond what static CXR can provide.*

**Supplementary information:**

The online version contains supplementary material available at https://doi.org/10.1007/s00431-026-07353-z.

## Introduction

Pulmonary complications are among the most frequent causes of morbidity and mortality after surgery for congenital heart disease, with a reported incidence of 6–76% [1]. Perioperative factors contributing to this high incidence include the systemic inflammatory response to cardiopulmonary bypass, surgical trauma near pleural surfaces, prolonged mechanical ventilation, and blood product transfusion. These collectively predispose pediatric patients to significant pulmonary dysfunction that directly prolongs mechanical ventilation and intensive care unit (ICU) stay [2].

Common postoperative pulmonary complications include atelectasis, pleural effusion, consolidation, pneumothorax, and diaphragmatic dysfunction [3]. While bedside chest X-ray (CXR) remains the traditional imaging reference, it delivers ionizing radiation and often misses early-stage pathology. Point-of-care lung ultrasound (LUS) has become a radiation-free, rapidly repeatable alternative that enables real-time monitoring of disease progression and therapeutic response [4]. Key sonographic signs, including B-lines, identify extravascular fluid accumulation in the early postoperative window [5].

The ESPNIC POCUS Working Group [6] formally endorses LUS as a first-line diagnostic and monitoring tool in critically ill neonates and children, citing high reproducibility, real-time capability, and absence of ionizing radiation. In congenital heart disease specifically, LUS detects subpleural consolidations, interstitial edema, and small effusions routinely missed by CXR, with direct implications for fluid management, diuretic titration, and physiotherapy decisions [4, 7, 8], and enables temporal monitoring of pulmonary recovery beyond what static radiography provides [5]. However, evidence remains predominantly derived from adult cohorts or small pediatric series, and rigorous prospective comparison of LUS with CXR across major complication categories and clinical outcomes remains lacking for children under 4 years undergoing congenital heart surgery.

Formal validation of LUS against conventional imaging in the immediate postoperative period remains limited in the pediatric cardiac surgery population. This pilot study therefore aimed to compare the detection rates and diagnostic agreement of LUS and CXR for pulmonary congestion, consolidation, pleural effusion, pneumothorax, and diaphragmatic motion abnormalities, and to explore potential predictors of extubation failure.

## Methods

### Study design and ethics

This prospective observational pilot study was conducted at the Pediatric Post-Cardiac Surgery Intensive Care Unit (PSICU), Cardiovascular Hospital, Faculty of Medicine, Ain Shams University, Cairo, Egypt (March 2024–March 2025). Ethical approval was granted by the Faculty of Medicine Research Ethics Committee (Ref. No.: FMASU MD155/2023; FWA 000017585). Written informed consent was obtained from the legal guardian of each participant. This work is adapted from an academic thesis submitted in partial fulfillment of the MD degree at the Faculty of Medicine, Ain Shams University; the thesis has not been published as a journal article elsewhere. This study is reported in accordance with the Strengthening the Reporting of Observational Studies in Epidemiology (STROBE) guidelines for cohort studies; a completed STROBE checklist is provided as Supplementary material.

### Participants

Thirty pediatric patients aged under 4 years undergoing congenital heart surgery were recruited by convenience sampling. Eligibility required RACHS-1 classification 1–3 to ensure a homogeneous low-to-moderate risk cohort. Exclusion criteria were pre-existing chronic respiratory disease, significant systemic comorbidities, or RACHS-1 category 4–6 surgery. Sample size was calculated using PASS 15 software, achieving statistical power > 99% for the primary comparison; however, the small total sample and limited extubation-failure subgroup (*n* = 5) restrict the power of secondary multivariable analyses, which should be interpreted as exploratory.

### Data collection

Baseline demographics, anthropometric data (interpreted via CDC growth charts), and continuous hemodynamic parameters were recorded including heart rate, systolic and diastolic blood pressure, central venous pressure (CVP), urine output, and fluid balance. Ventilatory parameters documented at each timepoint included peak inspiratory pressure, positive end-expiratory pressure (PEEP), tidal volume, and fraction of inspired oxygen (FiO_2_). Inotropic support was quantified using the Vasoactive-Inotropic Score (VIS), a validated composite measure of cardiovascular pharmacological burden in pediatric cardiac surgery [9]. Patients were assessed at three pre-specified timepoints: T1 (within 6 h postoperatively), T2 (pre-extubation), and T3 (within 12 h post-extubation). Timing was patient-specific (median time to T2: 16.0 h, IQR 5.0–24.5 h, range 2–291 h; median time to T3: 28.0 h, IQR 17.0–36.5 h). Daily assessments were also performed between T1 and T2. Comprehensive chest auscultation was documented at each interval.

### Lung ultrasound protocol

LUS was performed using a high-frequency linear probe. The standardized 12-zone protocol subdivided each hemithorax into six regions (anterior, lateral, and posterior, each further divided into superior and inferior quadrants) following Cantinotti et al. [4]. This 12-zone approach is consistent with the recent ESICM–ESPNIC international expert consensus on quantitative lung ultrasound [10] for children older than 12 months; the consensus recommends a modified five-region-per-hemithorax approach for younger infants, as detailed in the Discussion. Lung aeration was quantified using a validated Lung Ultrasound Aeration Score (range 0–36): each of the 12 regions was scored 0–3 using a coalescence-based approach (0, A-lines or ≤ 2 B-lines; 1, ≥ 3 well-spaced B-lines; 2, coalescent B-lines; 3, hepatization). The worst pattern per region was retained, consolidations of any size scored 3, and regions with limited access (tubes, drains, dressings) were scored from the visible portion. A high-frequency linear transducer was used in longitudinal intercostal orientation, patients positioned supine/semi-recumbent (lateral decubitus for posterior regions when feasible); B-mode was used for scoring, M-mode only to confirm sliding or pneumothorax. Machine settings (harmonics, artifact suppression) were not prospectively recorded, a noted limitation.

**Sonographic definitions:** (a) pulmonary congestion, ≥ 3 B-lines per intercostal space (coalescent B-lines); (b) consolidation, subpleural echo-poor region with or without air bronchograms (hepatization pattern); (c) pleural effusion, anechoic or hypoechoic space between parietal and visceral pleura (quad sign); (d) pneumothorax, absent lung sliding and B-lines with a barcode (stratosphere) sign on M-mode and preserved A-lines, with the lung point sought as confirmation where feasible; since absent sliding alone is non-specific (e.g., mainstem intubation, apnea, adhesions, hyperinflation, atelectasis), pneumothorax required this full constellation, and adjacent accessible spaces were used when tubes or dressings limited the window; (e) diaphragmatic dysfunction, reduced or paradoxical excursion on M-mode. These criteria are consistent with established ESPNIC point-of-care ultrasound guidelines [6]. As CXR does not directly visualize diaphragmatic motion, CXR-based DMA was inferred from static signs (elevated hemidiaphragm or reduced ipsilateral lung volume) a surrogate marker rather than a direct functional assessment, noted as a limitation.

**Operator experience:** LUS examinations were performed by two pediatric intensivists under the supervision of a diagnostic radiology consultant, each with a minimum of 2 years of point-of-care ultrasound experience and formal POCUS certification. Both operators completed a standardized training protocol based on published pediatric LUS guidelines [6] prior to study initiation. Inter-operator reliability was not formally assessed and constitutes a recognized limitation of this pilot study.

### Blinding

Strict blinding was maintained throughout the study: the LUS operator was blinded to CXR findings, and CXR was interpreted independently by an experienced radiologist blinded to LUS results.

### Statistical analysis

Quantitative variables are expressed as mean ± SD or median (IQR), categorical variables as frequencies and percentages. Group comparisons used the independent *t*-test and chi-square test (or Fisher’s exact test where cell counts were < 5). LUS–CXR agreement was assessed by Cohen’s kappa (0.00–0.20 slight; 0.21–0.40 fair; 0.41–0.60 moderate; 0.61–0.80 substantial); as kappa can be unstable at low prevalence, raw, positive, and negative percent agreement and discordant pairs are also reported (Supplementary Table S3). Given the number of comparisons across timepoints, these analyses are exploratory with no adjustment for multiplicity. Given the binary nature of the primary outcome (extubation failure vs. success), multivariable binary logistic regression was used to identify independent predictors, with results expressed as odds ratios (OR) with 95% confidence intervals. Due to the limited sample size (*n* = 30; failure subgroup *n* = 5), the logistic model was restricted to a maximum of two predictors to avoid overfitting, and all findings must be considered exploratory. Analyses used SPSS v23.0; *p* < 0.05 were considered statistically significant.

## Results

### Patient characteristics

**Fig. 1:**
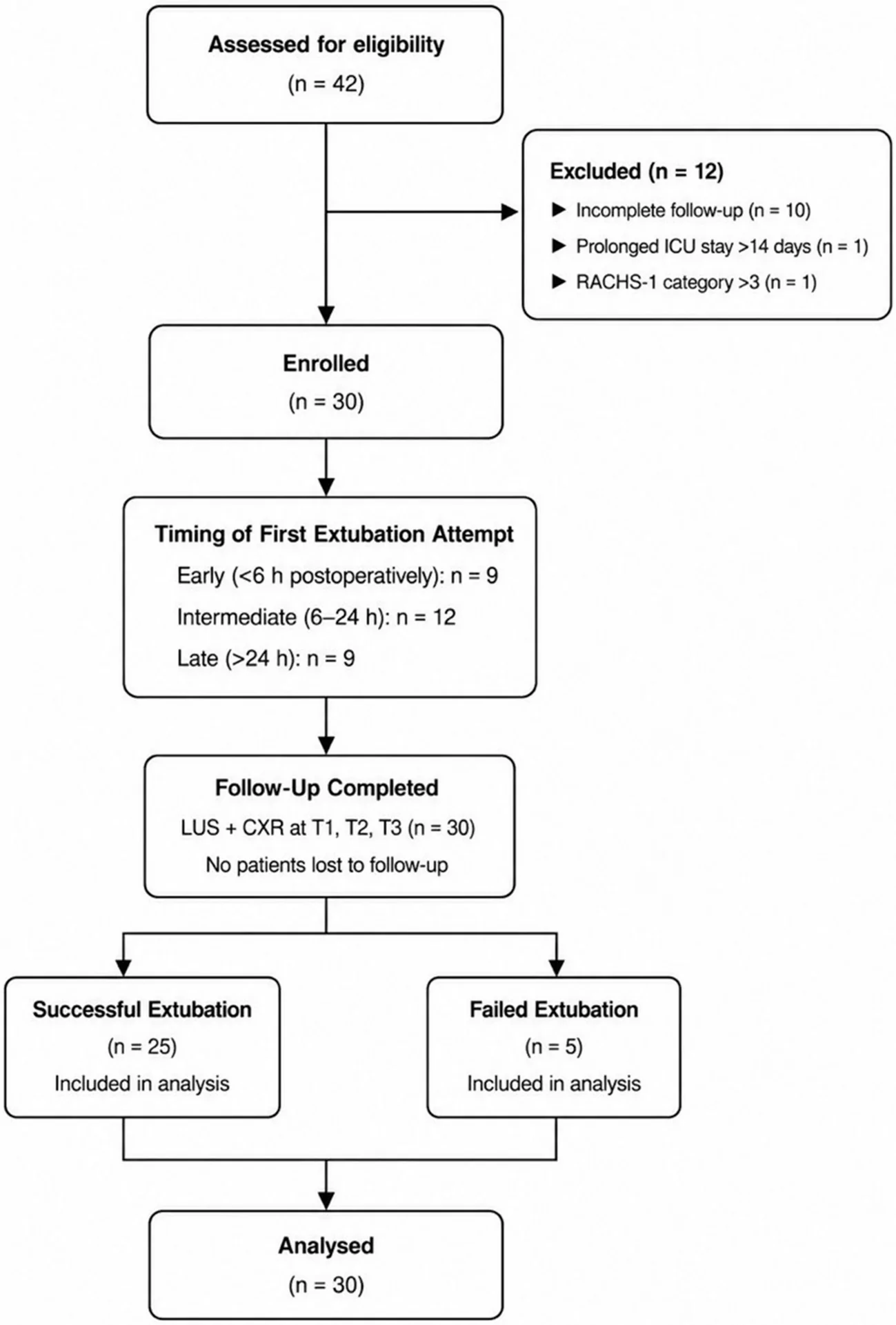
STROBE flow diagram. STROBE flow diagram of participant selection and follow-up. Of 42 patients assessed for eligibility, 12 were excluded (10 incomplete follow-up, 1 prolonged ICU stay > 14 days, 1 RACHS-1 category > 3), leaving 30 enrolled. Patients were stratified by timing of first extubation attempt (early < 6 h, *n* = 9; intermediate 6–24 h, *n* = 12; late > 24 h, *n* = 9). All 30 completed follow-up with no loss and were categorized into successful (*n* = 25) and failed (*n* = 5) extubation groups; all 30 were analyzed

Thirty patients were enrolled (mean age 18.0 ± 11.8 months; range 4–48 months; 60% male). Parental consanguinity was present in 13.3%. Reparative surgery was performed in 96.7% of cases. RACHS-1 categories 1, 2, and 3 comprised 20.0%, 63.3%, and 16.7% of the cohort, respectively (Table 1). All patients were managed within a dedicated 11-bed PSICU operating standardized postoperative protocols including continuous hemodynamic monitoring, goal-directed fluid management, and early physiotherapy. No patients were lost to follow-up, and all completed assessment at all three pre-specified timepoints (Fig. 1). Temporal trends in hemodynamic and physiological parameters over the 11-day postoperative period are illustrated in Supplementary Fig. S1.

**Table 1:** Baseline demographic and surgical characteristics of the study cohort (*n* = 30)

Characteristic	Value
Age (months), mean ± SD (range)	18.0 ± 11.8 (4–48)
Sex: male/female, *n* (%)	18 (60.0%)/12 (40.0%)
Consanguinity, *n* (%)	4 (13.3%)
Surgical type, *n* (%)	
Reparative	29 (96.7%)
Palliative	1 (3.3%)
RACHS-1 category, *n* (%)	
Category 1	6 (20.0%)
Category 2	19 (63.3%)
Category 3	5 (16.7%)

*RACHS-1* risk adjustment for congenital heart surgery. Data are expressed as mean ± SD (range) or *n* (%)

### Comparative LUS and CXR detection rates

**Fig. 2:**
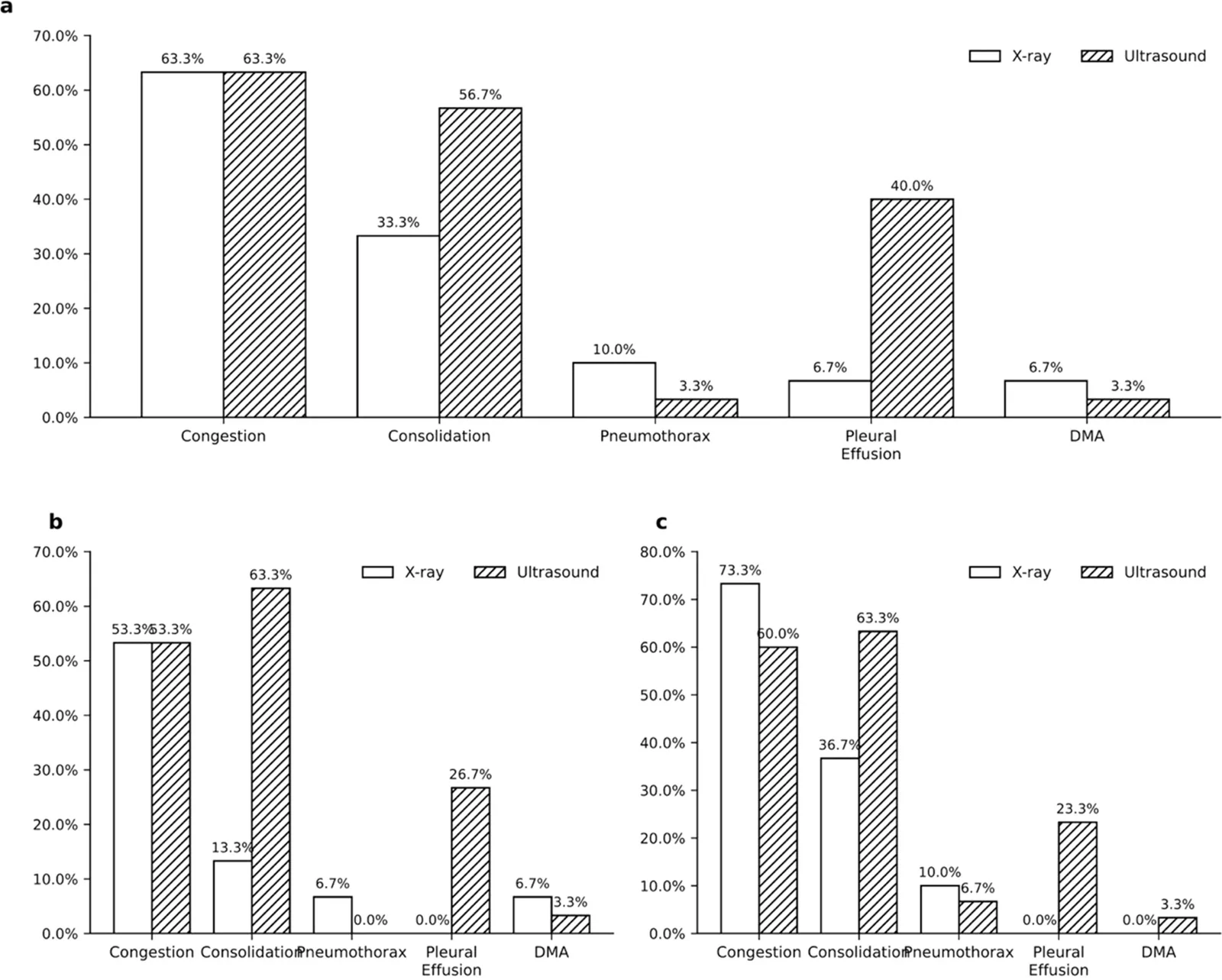
Detection rates (%) of pulmonary complications by LUS (hatched bars) and CXR (open bars). Assessed at **a** T1 (0–6 h postoperatively), **b** T2 (pre-extubation), and **c** T3 (post-extubation). No reference standard was used; values represent detection rates only. DMA, diaphragmatic motion abnormalities

Results at all three assessment timepoints (T1, 0–6 h; T2, pre-extubation; T3, post-extubation) are presented in Table 2 and Fig. 2. As no CT or other reference standard was used, findings are reported as detection rates and inter-modality agreement rather than measures of diagnostic accuracy. At T1, LUS and CXR detected equivalent rates of pulmonary congestion (63.3%; *p* = 1.000; kappa = 0.426). LUS detected higher rates of consolidation (56.7% vs. 33.3%; *p* = 0.069; kappa = 0.426) and pleural effusion (40.0% vs. 6.7%; *p* = 0.002; kappa = 0.194). At T2, LUS detected substantially more consolidation (63.3% vs. 13.3%; *p* < 0.001; kappa = 0.164, slight agreement) and pleural effusion (26.7% vs. 0%; *p* = 0.002; kappa = 0.000), with substantial agreement for congestion (kappa = 0.732). Pneumothorax and diaphragmatic motion abnormalities were infrequent at all timepoints; no significant inter-modality differences were observed for these parameters (all *p* > 0.05).

**Table 2:** Comparison of LUS and CXR detection rates and diagnostic agreement across all assessment timepoints

Complication/modality	T1, 0–6 h CXR/LUS, *n* (%)	*p*-value	*κ* (95% CI)	T2, pre-extubation CXR/LUS, *n* (%)	*p-value*	*κ* (95% CI)	T3, post-extubation LUS, *n* (%)
Pulmonary congestion							
CXR	19 (63.3%)	1.000	0.426 (0.09–0.76)	16 (53.3%)	1.000	0.732 (0.49–0.98)	22 (73.3%)
LUS	19 (63.3%)	—	—	16 (53.3%)	—	—	18 (60.0%)
Pulmonary consolidation^a^							
CXR	10 (33.3%)	0.069	0.426 (0.14–0.71)	4 (13.3%)	< 0.001*	0.164 (0.00–0.33)	11 (36.7%)
LUS	17 (56.7%)	—	—	19 (63.3%)	—	—	19 (63.3%)
Pleural effusion^b^							
CXR	2 (6.7%)	0.002*	0.194 (0.05–0.44)	0 (0.0%)	0.002*	0.000	0 (0.0%)
LUS	12 (40.0%)	—	—	8 (26.7%)	—	—	7 (23.3%)
Pneumothorax							
CXR	3 (10.0%)	> 0.05	—	2 (6.7%)	> 0.05	—	2 (6.7%)
LUS	1 (3.3%)	—	—	0 (0.0%)	—	—	1 (3.3%)
Diaphragmatic motion abnormalities (DMA)							
CXR	2 (6.7%)	> 0.05	—	1 (3.3%)	> 0.05	—	1 (3.3%)
LUS	1 (3.3%)	—	—	1 (3.3%)	—	—	1 (3.3%)

T1 = 0–6 h postoperatively; T2 = pre-extubation; T3 = post-extubation. *p*-values by McNemar test. *p < 0.05. *κ* = Cohen’s kappa (95% CI): 0.00–0.20 slight, 0.21–0.40 fair, 0.41–0.60 moderate, 0.61–0.80 substantial. No reference standard (CT) was used; values represent detection rates only. (a) Consolidation, subpleural echo-poor region ± air bronchograms (hepatization pattern). (b) Pleural effusion, anechoic/hypoechoic space between pleural layers (quad sign). *LUS* lung ultrasound, *CXR* chest X-ray, *CI* confidence interval, *DMA* diaphragmatic motion abnormalities

Composite LUS aeration scores paralleled these detection-rate findings: the total score (0–36 scale) was numerically higher at all three timepoints in patients who subsequently failed extubation, reaching significance at T1 (median 22.0 [IQR 21.0–22.0] vs. 17.0 [14.0–21.0]; *p* = 0.047) but not at T2 or T3 (Supplementary Table S4).

### Extubation outcomes and predictors

Extubation failure occurred in 16.7% (*n* = 5). Successful extubation was associated with fair bilateral air entry on auscultation (78.3% vs. 20.0%; *p* = 0.019) and shorter inotropic support duration (2.65 ± 2.21 vs. 5.50 ± 3.11 days; *p* = 0.040). At T2, LUS detected consolidation in all failed cases (100%) vs. 60.9% of successes (*p* = 0.036), whereas CXR showed no significant difference (40.0% vs. 9.1%; *p* = 0.079). LUS detected pleural effusion appeared only in successful cases (34.8% vs. 0%; *p* = 0.050), suggesting small effusions did not impair weaning in this cohort. Extubation failure was associated with significantly worse outcomes including prolonged ICU stay (45.2 vs. 5.28 days; *p* < 0.001), longer ventilation (15.01 ± 20.07 vs. 1.08 ± 1.81 days; *p* = 0.001), and higher mortality (20.0% vs. 0%; *p* = 0.005; Table 3). Given the very small failure subgroup (*n* = 5), all comparisons must be interpreted with caution.

**Table 3:** Clinical and radiological predictors of extubation outcome

Variable	Failed extubation (*n* = 5)	Successful extubation (*n* = 25)	p
Clinical parameters — chi-square/independent *t*-test			
Fair air entry bilateral, *n* (%)	1 (20.0%)	18 (78.3%)	0.019*
Inotropic support duration (days), mean ± SD	5.50 ± 3.11	2.65 ± 2.21	0.040*
LUS findings — pre-extubation (T2), *n* (%) Fisher’s exact test			
Pulmonary consolidation^a^	5 (100.0%)	14 (60.9%)	0.036*
Pulmonary congestion	3 (60.0%)	13 (56.5%)	0.887
Pleural effusion^b^	0 (0.0%)	8 (34.8%)	0.050
CXR findings — pre-extubation (T2), *n* (%) chi-square test			
Pulmonary consolidation	2 (40.0%)	2 (9.1%)	0.079
Pulmonary congestion	4 (80.0%)	12 (52.2%)	0.254
Postoperative outcomes — independent *t*-test/Fisher’s exact test			
ICU length of stay (days), mean ± SD	45.2 ± 31.36	5.28 ± 4.55	< 0.001*
Total ventilation duration (days), mean ± SD	15.01 ± 20.07	1.08 ± 1.81	0.001*
Mortality, *n* (%)	1 (20.0%)	0 (0.0%)	0.005*

*p < 0.05. Statistical tests indicated in each section header. Fisher’s exact test used where cell count < 5. Due to the small failure subgroup (*n* = 5), all comparisons should be interpreted with caution. (a) Hepatization pattern. (b) Quad sign. *LUS* lung ultrasound, *CXR* chest X-ray, *ICU* intensive care unit

### Multivariable logistic regression

To explore independent predictors of extubation failure, a binary multivariable logistic regression model was constructed with inotropic support duration and LUS consolidation as candidate predictors (restricted to two variables given *n* = 5 failures). The OR for inotropic support duration (OR 0.52; 95% CI 0.32–0.85; *p* = 0.009; Supplementary Table S1) reflects that higher inotropic support duration is associated with lower probability of successful extubation, consistent with greater hemodynamic instability in failed cases. LUS consolidation did not reach statistical significance (OR 2.10; 95% CI 0.56–7.89; *p* = 0.274); notably, consolidation was present in 100% of failed extubations, raising the possibility of quasi complete separation and further limiting the stability of this estimate. Given only five failure events, this model is markedly underpowered and at substantial risk of overfitting; results should be regarded as strictly exploratory and hypothesis generating rather than evidence of independent predictors, and require confirmation in adequately powered, ideally multicenter, studies.

## Discussion

This prospective pilot study compared LUS and CXR detection rates in 30 pediatric patients following congenital heart surgery. LUS detected higher rates of pleural effusion and consolidation than CXR at multiple timepoints, while both showed equivalent congestion detection. As no reference standard (e.g., CT) was used, findings reflect inter-modality detection differences rather than diagnostic accuracy, warranting cautious interpretation.

At T1, LUS detected consolidation (56.7% vs. 33.3%) and effusion (40.0% vs. 6.7%) more often than CXR. Bajracharya et al. [11] reported 90% LUS sensitivity and 98% negative predictive value for pleural effusion detection in a comparable cohort. Higher LUS detection rates may partly reflect thinner pediatric chest walls facilitating identification of subpleural pathology missed by CXR [9]. These observations are consistent with Cantinotti et al. [4] and Raimondi et al. [12], who demonstrated that LUS identifies consolidations and small effusions invisible on conventional radiography and may directly alter clinical management [13].

Inter-modality agreement was substantial for congestion at T2 (kappa = 0.732) but only slight for consolidation (kappa = 0.164), reflecting known overlap between consolidation, atelectasis, and subpleural pathology [14]. The exclusive detection of pleural effusion in the successful extubation group by LUS (34.8% vs. 0%) suggests small effusions did not adversely affect weaning here, consistent with Shrestha et al. [15]. These discrepancies highlight the need to integrate imaging with broader clinical judgment.

LUS pneumothorax detection was numerically lower than CXR (3.3% vs. 10.0%), likely reflecting technical limitations and operator-dependent LUS performance in this setting [6].

On multivariable logistic regression, longer inotropic support duration was associated with higher odds of extubation failure (*p* = 0.009), while imaging variables were not independently significant. This exploratory finding is consistent with prior reports emphasizing the primacy of hemodynamic stability over isolated imaging findings in weaning decisions [7, 16, 17]. However, given the failure subgroup of only five patients, this model is severely underpowered, and these results are purely hypothesis generating. Traditional static parameters including IVC indices and CVP were also non-predictive, consistent with Sée et al. [18]. Inotropic support duration, as recorded, reflects total postoperative duration and may partly extend beyond the extubation attempt including any reintubation period and so may partly reflect a consequence of failure and overall severity rather than a strictly pre-extubation predictor; pre-extubation restricted variables (e.g., VIS, vasoactive status, lactate) would be preferable and merit priority in future studies. ICU stay, ventilation duration, and mortality are reported as outcomes, not predictors.

**Fig. 3:**
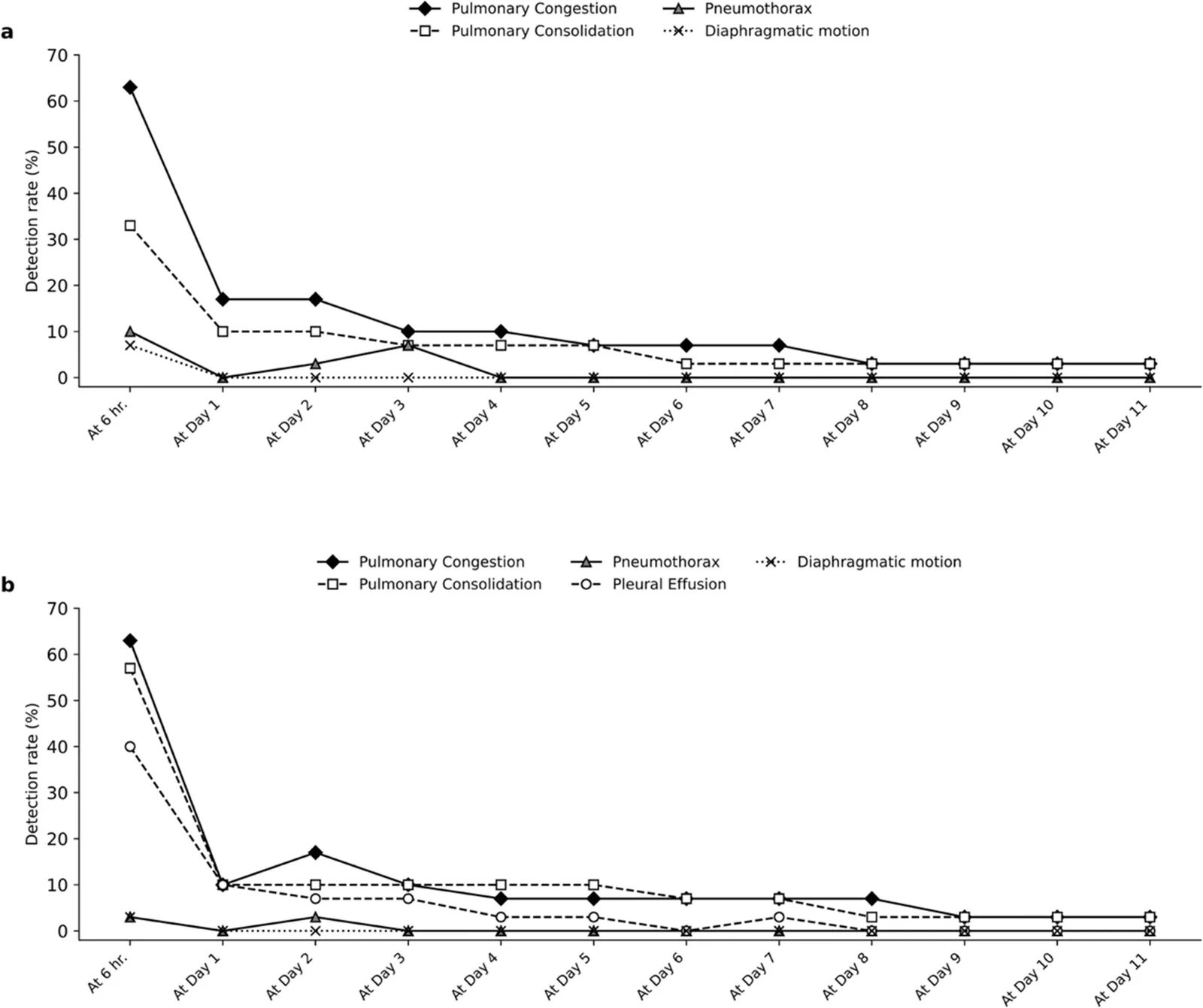
Temporal trends in detection rates of postoperative pulmonary complications over 11 days. As detected by **a** CXR and **b** LUS

Beyond this diagnostic comparison, LUS identified pleural effusion and consolidation missed by CXR at multiple timepoints and enabled temporal tracking of complication resolution over 11 days (Fig. 3), supporting its role as a complementary, longitudinal monitoring tool alongside CXR [6] for fluid management, weaning, and physiotherapy timing. As POCUS adoption grows [4, 7], standardized training and validated scoring will be essential to reduce operator-dependent variability, an acknowledged limitation here.

This study has several important limitations. First and most critically, the sample of 30 patients with only five extubation failures severely limits the statistical power of all subgroup analyses and precludes definitive conclusions regarding predictors of extubation failure. Second, the single-center, convenience-sampled design limits generalizability. Third, restriction to RACHS-1 categories 1–3 means findings may not apply to higher complexity surgical cases. Fourth, the absence of CT imaging as a reference standard prevents determination of true diagnostic accuracy; our findings describe detection rates and agreement only. Fifth, inter-operator reliability was not assessed. Sixth, the use of convenience rather than consecutive sampling may introduce selection bias, and the 12-month study period may not capture seasonal variation in infection-related pulmonary complication rates. Seventh, per the recent ESICM–ESPNIC international expert consensus on quantitative lung ultrasound [10], which we have adopted for terminology (“LUS aeration score”) and scoring approach (Methods), region selection differs by age: a 12-region protocol (six per hemithorax) is endorsed for children ≥ 12 months, consistent with our protocol [4] and applicable to 19 of our patients, whereas infants under 12 months warrant a modified five-region-per-hemithorax approach (10 zones total) [19]. We applied the 12-zone protocol uniformly, including in the 11 of 30 patients (36.7%) younger than 12 months, which does not fully align with this age-specific recommendation. We acknowledge this as a limitation rather than grounds to discard the affected data. A post hoc sensitivity analysis (Supplementary Table S2) confirmed the composite LUS score was significantly higher in this younger subgroup (mean 21.4 vs. 15.7, *p* = 0.003), likely reflecting protocol-related score inflation rather than a true complication burden difference, while individual complication detection rates did not differ significantly by age group (all *p* > 0.05), supporting the strength of the primary findings. Finally, ultrasound machine settings (harmonics, artifact suppression) were not prospectively recorded, so constancy across examinations cannot be confirmed; although the same trained operator used a consistent protocol, a contribution of unrecorded settings cannot be fully excluded, and future prospective studies should record these explicitly. Future multicenter, prospective studies with larger, adequately powered samples and a CT reference standard are needed to validate these preliminary observations.

## Conclusion

In this single-center pilot study of 30 pediatric patients, LUS demonstrated higher detection rates for early postoperative pleural effusion and consolidation compared with CXR, supporting its role as a complementary bedside imaging tool in pediatric cardiac surgery. However, given the small sample size and single-center design, these findings are preliminary and should not be used as the basis for definitive clinical practice recommendations without confirmation in larger prospective studies. Extubation outcomes appeared primarily associated with hemodynamic stability specifically the duration of inotropic support rather than imaging findings alone, reinforcing the need for an integrated clinical approach to postoperative management.

## Supplementary information

Below is the link to the electronic supplementary material.

ESM 1 (PNG 642 KB)

ESM 1 High Resolution Image (TIF 768 KB)

ESM 2 (DOCX 21.3 KB)

## Data Availability

The datasets generated and/or analyzed are available from the corresponding author on reasonable request.
