# Supplementary material for: Lung ultrasound versus chest radiography for the detection of early postoperative pulmonary complications in children undergoing cardiac surgery
Source: Eur J Pediatr. 2026 Sep 9;185(10):731. doi: 10.1007/s00431-026-07353-z (PMC13558279; doi:10.1007/s00431-026-07353-z)
Supplement: Supplementary file 1 [file 431_2026_7353_MOESM2_ESM.docx]

**Supplementary Material**

*Lung Ultrasound versus Chest Radiography for the Detection of Early Postoperative Pulmonary Complications in Children Undergoing Cardiac Surgery*

Bakry NA, Mansour MG, Ali HA, Abouelhoda AM, Abd Elhamid MI, Ebrahim BA

*European Journal of Pediatrics*

**Supplementary Fig. S1 legend**

Temporal evolution of postoperative hemodynamic and physiological parameters.

**Panel a:** Trends in vital signs: heart rate (HR), systolic blood pressure (SBP), diastolic blood pressure (DBP), and body temperature from 6 hours postoperatively through Day 11. HR was markedly elevated at 6 hours and progressively declined toward baseline by Day 5. SBP and DBP exhibited early variability with gradual stabilization; SBP consistently exceeded 100 mmHg from Day 4 onward. Body temperature remained within the normal physiological range throughout.

**Panel b:** Trends in central venous pressure (CVP; mmHg), urine output (UOP; mL/kg/hr), and fluid balance (FB; numerical value). CVP peaked on Day 4 and subsequently stabilized. Fluid balance transitioned from positive to a sustained negative state from Day 3 onward, coinciding with urine output exceeding 4 mL/kg/hr collectively reflecting successful postoperative diuresis and fluid offloading.

**Supplementary Table S1**

**Table S1.** Multivariable binary logistic regression exploratory analysis of factors associated with extubation failure.

| **Variable** | **OR** | **SE** | **95% CI** | **p-value** |
| --- | --- | --- | --- | --- |
| **Primary Clinical Predictors** | | | | |
| Inotropic support duration (days) | 0.52 | 0.18 | 0.32–0.85 | 0.009* |
| Age (months) | 1.04 | 0.02 | 0.99–1.08 | 0.060 |
| **Imaging Variables — Pre-extubation (T2)** | | | | |
| LUS consolidation | 2.10 | 1.45 | 0.56–7.89 | 0.274 |
| LUS pleural effusion | 0.88 | 0.62 | 0.18–4.31 | 0.872 |
| **Outcome Variable** | | | | |
| Total ventilation duration (days) | 0.91 | 0.07 | 0.79–1.04 | 0.152 |

** p<0.05. Binary logistic regression used (binary outcome). Model restricted to two predictors due to small failure subgroup (n=5); all results are exploratory. OR = odds ratio (OR <1 indicates lower odds of failure); SE = standard log-OR error; CI = confidence interval. LUS = Lung Ultrasound.*

**Supplementary Table S2**

**Table S2.** Sensitivity analysis of the 12-zone LUS protocol in patients under 12 months of age.

The 12-zone LUS protocol applied in this study aligns with ESICM–ESPNIC consensus recommendations for children ≥12 months of age; a modified 10-zone protocol is recommended for younger infants. Of the 30 study patients, 11 (36.7%) were under 12 months (range 4–11 months). This analysis examines whether protocol mismatch affected study findings in this subgroup.

**A. Composite LUS score**

| **LUS Score (T1, 0–6h)** | **<12 months (n=11)** | **≥12 months (n=19)** |
| --- | --- | --- |
| Mean ± SD | 21.36 ± 4.99 | 15.74 ± 4.33 |
| Range | 10–29 | 7–22 |
| Independent t-test | t = 3.25, p = 0.003* | — |

** p<0.05. The composite LUS score (12-zone scale, range 0–36) was significantly higher in patients <12 months, most plausibly reflecting protocol-related score inflation rather than a true difference in pulmonary complication burden.*

**B. Individual complication detection rates (T1, 0–6h)**

| **Finding (T1, 0–6h)** | **<12 mo (n=11)** | **≥12 mo (n=19)** | **Fisher p** |
| --- | --- | --- | --- |
| Pulmonary congestion | 7 (63.6%) | 12 (63.2%) | 1.000 |
| Consolidation | 8 (72.7%) | 9 (47.4%) | 0.259 |
| Pleural effusion | 3 (27.3%) | 9 (47.4%) | 0.442 |
| Pneumothorax | 1 (9.1%) | 0 (0.0%) | 0.367 |
| Diaphragmatic motion abnormality | 1 (9.1%) | 0 (0.0%) | 0.367 |

*Detection rates for each individual sonographic finding did not differ significantly between age groups (all Fisher's exact p>0.05), indicating the qualitative diagnostic conclusions are not materially confounded by the age-related protocol mismatch.*

**Supplementary Table S3**

**Table S3.** LUS–CXR raw, positive, and negative percent agreement at T2 (pre-extubation), supplementing Cohen's kappa reported in Table 2.

| **Finding (T2)** | **n** | **Raw Agreement** | **Pos. Agreement** | **Neg. Agreement** | **Discordant** | **κ** |
| --- | --- | --- | --- | --- | --- | --- |
| Congestion | 28 | 85.7% | 77.8% | 71.4% | 4 | 0.708 |
| Consolidation | 26 | 46.2% | 22.2% | 36.4% | 14 | 0.150 |
| Pleural Effusion | 27 | 70.4% | 0.0% | 70.4% | 8 | 0.000 |
| Pneumothorax | 27 | 96.3% | 0.0% | 96.3% | 1 | 0.000 |
| DMA | 23 | 95.7% | 50.0% | 95.5% | 1 | 0.646 |

*Positive agreement = agreement among cases where at least one modality was positive; negative agreement = agreement among cases where at least one modality was negative. Kappa may be unstable or undefined (0.000) when prevalence is very low or when one modality detects no events, as reflected by the divergence between raw and chance-corrected agreement for pneumothorax and pleural effusion. DMA = Diaphragmatic Motion Abnormality.*

**Supplementary Table S4**

**Table S4.** Serial total Lung Ultrasound Aeration Scores (0–36 scale) across perioperative timepoints, by extubation outcome.

| **Timepoint** | **Overall (N=30) Median [IQR]** | **Successful (n=25) Median [IQR]** | **Failed (n=5) Median [IQR]** | **p*** |
| --- | --- | --- | --- | --- |
| 0–6 h postoperative (T1) | 19.0 [15.5–22.0] | 17.0 [14.0–21.0] | 22.0 [21.0–22.0] | **0.047*** |
| Pre-extubation (T2) | 19.0 [14.3–21.0] | 19.0 [14.0–20.0] | 21.0 [19.0–22.0] | 0.124 |
| Post-extubation (T3) | 20.0 [15.3–23.0] | 19.0 [15.0–23.0] | 23.0 [21.0–24.0] | 0.200 |

** Mann-Whitney U test, Successful vs. Failed extubation. The composite score was significantly higher in patients who failed extubation at T1, but not at T2 or T3, consistent with the individual detection-rate findings in Table 3.*
